# Translational Research in the Time of COVID-19—Dissolving Boundaries

**DOI:** 10.1371/journal.ppat.1008898

**Published:** 2020-09-30

**Authors:** Jonathan D. Edgeworth, Rahul Batra, Gaia Nebbia, Karen Bisnauthsing, Eithne MacMahon, Malur Sudhanva, Sam Douthwaite, Simon Goldenberg, Geraldine O’Hara, Manu Shankar-Hari, Katie J. Doores, Rocio Martinez-Nunez, Carolyn Hemsley, Nicholas M. Price, Jill Lockett, Robert I. Lechler, Stuart J. D. Neil, Michael H. Malim

**Affiliations:** 1 Centre for Clinical Infection and Diagnostics Research, Department of Infectious Diseases, School of Immunology and Microbial Sciences, King’s College London, London, United Kingdom; 2 Department of Infectious Diseases, Guy’s and St Thomas’ NHS Foundation Trust, London, United Kingdom; 3 Viapath, London, United Kingdom; 4 Department of Virology, King’s College Hospital, London, United Kingdom; 5 King’s Health Partners, London, United Kingdom; University of Pittsburgh, UNITED STATES

Scientific research gathers data and evidence to test hypotheses within a solid almost philosophical framework that has evolved over hundreds of years [1]. About 30 years ago, the concept of translational research emerged across life sciences, first appearing in PubMed in 1993, to support the increased flow of scientific discovery through to patient and societal benefit [2]. It was cemented as a strategic priority in the United Kingdom about 10 years later with the creation of nationally commissioned Biomedical Research Centres (BRCs). Different steps in the path from ‘bench-to-bedside’ were defined moving through proof of principle, defining mechanisms, evaluating benefits, piloting, large trials, outcome-informed improvements, and adoption into the healthcare system. Designated funding streams were created to support each stage, encouraging researchers to balance their distinctive contributions and establish transparent criteria to inform facile progression to the next step of the pathway. Taken together, the overarching objective has been to optimally balance the pursuit of intellectual brilliance with cost-effective and rapid flow from discovery to delivery.

From a frontline perspective of clinical infectious diseases, clinical virology, infection prevention and control, laboratory diagnostic service, and translational research, Coronavirus Disease 2019 (COVID-19)/Severe Acute Respiratory Syndrome Coronavirus 2 (SARS-CoV-2) has turned our world upside down. This is a personal account of that impact from within a UK Academic Health Sciences Centre (AHSC), King’s Health Partners (KHP) [3], after about 4 months observing colleagues coming together in response to a new and acutely dangerous disease. Like many academically aligned healthcare institutions around the world, we have seen a large cohort of patients in a short space of time; for us, approximately 3,200 patients of whom about 800 spent time in an intensive care unit. Here, we draw out some potentially common themes that emerged on our journey to introducing and scaling up delivery and development of novel diagnostic tests and services whilst also recruiting patients into clinical trials, commencing disease pathogenesis research projects, contributing data and samples for nationally coordinated projects, and responding to rapidly changing clinical service needs, presumably an agenda that colleagues in analogous organisations around the world will be facing. Our focus is on how clinicians, clinical academics, research and healthcare scientists as well as research nurses came together on the ground to highlight the successes and opportunities alongside the challenges and obstacles that may well be common themes.

## Setting up a COVID-19 diagnostics and translational research group

### Who are we?

(1) King’s College London research scientists with expertise in virology, molecular biology, and human immunology on an ambulatory and elective surgery site juxtaposed with the central hub of our comprehensive BRC (Guy’s Hospital) [4]; (2) ISO15189-accredited diagnostic bacteriology and virology laboratories run by our pathology provider, Viapath [www.viapath.co.uk], on 2 acute care academic hospital sites (St Thomas’ and King’s College Hospitals); (3) clinical virology and infectious diseases departments on both acute hospital sites with, relevant to COVID-19, a translational diagnostics research group adjacent to the diagnostic laboratories and a nationally commissioned centre for respiratory high consequences infectious diseases. As a result, like many multisite healthcare organisations, people and resources were located on different sites managed by different sovereign organisations, each about 1 mile apart. These interorganisational, geographical, and professional boundaries presented familiar challenges to collaboration and partnership working. None of us had a coronavirus research background, nevertheless, 1 unifying theme for each group was having wet laboratory activity as part of their contribution to the diagnostics pathway.

### What happened at the start?

Things largely materialised more than being consciously planned. A single vision emerged shared by all staff and students to pool expertise and resources ‘at a moment in time’ to respond to this new largely unknown deadly disease. This blurred the boundaries between our prior missions of research and education, laboratory service provision, and patient care. Small teams coalesced initially through prior personal contact but within a few weeks into a single consortium. It became clear that the best way of working required us to share openly, engage with agreed common goals, undertake new tasks, and take risks—all whilst working in unfamiliar territories.

The initial focus was introducing and scaling up new diagnostics at pace with service resilience for anticipated increases in demand and reagent shortages [5,6]. The core laboratory output was 2 main tests: 1 for pathogen detection, largely SARS-CoV-2 RNA in nose and throat swabs using initially quantitative reverse transcription polymerase chain reaction (RT-qPCR) and a host biomarker of infection, namely serum antibodies. These tests linked with multiple translational research needs and opportunities: developing and evaluating novel protocols or technologies; understanding the pathogenesis of coronavirus infection; supporting changing demand in hospitals; community asymptomatic SARS-CoV-2 RNA screening and serological testing for staff, students, and the wider population for public health benefit; and supporting recruitment of patients into clinical trials principally of novel therapeutics and vaccines.

A weekly diagnostics expansion meeting was established with senior representation from each KHP sovereign organisation alongside commissioners and public health, hosted by the KHP managing director. This provided support for procurement, logistics, and communication within a single operational framework, and critically, leadership consistently made the whole effort feel inclusive, with all parties being valued and engaged.

### What was in place to facilitate this new way of working?

Progress over the past 10–15 years had created a shared recognition of the value of evidence-based approaches and translational research. Particularly important were (1) honorary university appointments for National Health Service (NHS) medical and allied healthcare professional staff, where clinical impact and a more inclusive view of scholarship were given credit alongside traditional publication and grant-holding metrics; (2) seed-funding for posts and core facilities managed through the university and our BRC; and (3) Integrated Academic Training programmes for clinical and scientific staff undertaking PhDs and Academic Clinical Fellowships, an important enabler for the mobility of ideas and career paths between each world. Together, this provided a shared understanding to enable delivery through the weekly diagnostics expansion group.

## Identifying behavioural success factors

Everyone contributed their time, expertise, and resources to that single purpose, working in the same laboratories, offices, or e-mail groups, helping each other outside their usual domain whether that was supporting research, diagnostic development, or service delivery. Three principles emerged:

### Everyone together from the start

Everyone was invited to meetings to plan, make decisions, and resolve issues and share the success from original discovery to final patient impact. Workflows were iterative and often with different elements running in parallel rather than sequential, such as basic science experiments, writing draft validation and evaluation protocols of multiple potential technologies, or talking to users about their needs to write draft service guidelines. People working on late-stage projects were therefore content to accept their work was at risk if 1 component failed or changed at an earlier stage.

### Sharing each other’s roles

Research scientists mothballed previous projects to work on other colleagues’ research projects, contributing to the writing of training and competency assessments for delivering diagnostic services, sometimes from their own repurposed research facilities; healthcare scientists contributed to and adopted novel technologies and ideas led by university scientists; clinicians and research nurses with access to diagnostic laboratories embraced the collection and archiving of (longitudinal) blood samples from patients and staff, linking with important clinical data before anonymisation for use by members of the consortium or submission to national and international bodies (e.g., International Severe Acute Respiratory and Emerging Infection Consortium (ISARIC)).

### Understanding and appreciating each other’s skills, experience, and professional framework

Everyone found out what goes on in each other’s worlds, many perhaps for the first time—their priorities, aspirations, and questions. We started to break down entrenched unhelpful professional stereotypes, highlighting common transferable laboratory skills between research and service, learning that each has strong professional commitment, rigour, and clarity of thought but just in a (formerly) different conceptual framework. Terms like ‘soup-to-nuts’, ‘bench-to-bedside’, and the enduring importance of cooler conversations—COVID-19 brought them to life as never before. It embodied the founding spirit of our AHSC, but it took a pandemic to make it real.

## What progress has been made in the first 4 months?

Our assessment highlights (at least) 4 areas of thriving translational research: (1) ‘delivering and adapting the clinical service’—caring for COVID-19 patients on an infectious diseases ward whilst providing a consult and infection prevention and control service across the hospitals. This gave visibility of core and rapidly changing clinical needs; (2) ‘developing laboratory diagnostic capability’—setting up a new COVID-19 diagnostic expansion laboratory embedded within academic research facilities at Guy’s Hospital introducing novel accredited pipelines; technologies; PCR or serological tests; and COVID-19 or metagenomic sequencing for clinical service, infection control, and public health asymptomatic screening [5–8] (we named this new laboratory in recognition of June Almeida who discovered coronaviruses by electron microscopy at St Thomas’ Hospital in 1966 [9]); (3) ‘conducting fundamental research’—comprehensive immunophenotyping to understand correlates of disease/protection and to inform novel therapies; virus culture to dissect viral replication and support preclinical drug discovery; and viral sequencing to inform transmission networks, immune escape, and persistence [10–12]; (4) ‘supporting therapeutics and vaccine research’—recruiting patients into nationally coordinated clinical trials of new therapies (e.g., convalescent serum, vaccines, and biologics) and participating in cohort-based and population studies to explore, inter alia, rates of infection, disease severity risk factors, and renewed or persistent COVID-19 infection [13].

It is premature to reach conclusions on the impact and outputs of these new working arrangements, although 2 early examples are worth mentioning. First, within 3 months we moved from investigating antibody responses to COVID-19 infection and the evaluation of over 10 different diagnostic technologies to setting up a first pilot patient serology service to identify the clinical benefit of antibody detection for managing suspected post-COVID-19 syndromes and persistent infection [7,8,10]. This would have been inconceivable before COVID-19: a single team worked across the pathway from start to finish including, e.g., university scientists being on the daily serology service rota. Second, our King’s College Hospital laboratory was at the forefront of NHS community screening in testing all residents and staff in 50 care homes across South East London boroughs and formed much of the basis for a submission of a large KHP bid for scaled up and linked hospital, key worker and community (symptomatic and asymptomatic) testing, and surveillance capability in South East London.

## Forces likely to drive us apart when this pandemic is over

As society and healthcare institutions tentatively seek to return to normality, we can see that this new way of working is fragile with forces pulling us back. First, furlough, national underwriting of resources required to fight the pandemic and the willingness of finance in each organisation to temporarily accept the clinical need, meant financial incentives aligned with the partnership approach. This will not last when organisations start to realign around their previous funding models. A new more integrated strategic business plan will be required to keep this together, demonstrating convincingly to many stakeholders why this is better than past financial and strategic models that have engendered a siloed outlook.

Secondly, the parallel professional hierarchies within and between academic research, clinical service, and laboratory service: between research and healthcare scientists, full-time clinicians, and academic clinicians. These were exposed when we came together requiring some honest and sometimes difficult conversations to build mutual respect. The creation of a local multi-professional grouping committed to a shared endeavour with daily interactions felt like the primary delivery vehicle for diagnostic translational researchers taking innovation through to patient impact. Without continued support working across professional boundaries, including more flexible career paths and an integrated professional framework for healthcare and research scientists, articulated through a more realistic set of metrics of success and performance, the forces of familiarity will take us back.

Thirdly, wet laboratory consolidation strategies have dominated the direction of travel for the past 30 years often built around large technology platforms, be that for NHS pathology services, discovery science, public health, or national centres of excellence such as for genomics. This introduces additional geographical boundaries between members of a multi-professional team. Our ability to respond to this crisis would have been much harder had our diagnostic laboratories not been embedded in our clinical services, and we reflect on what more might have been achieved if university scientists had also been colocated.

National initiatives often within 1 professional body typically endorse the establishment of large consortia, national sample collections, centralised pathology, or public health laboratory hubs as well as small numbers of nationally commissioned centres of laboratory research excellence as lead delivery vehicles, including in this crisis. This approach clearly works as a model for advancement in therapeutics, vaccines, and human genetics. Yet the challenge translating novel diagnostics into NHS service has been noted for many years, and the UK SARS-CoV-2 testing response has attracted considerable criticism, in both cases comparing unfavourably with advancement in therapeutic innovation. The successful translational research model may be different for diagnostics, and additionally so for infectious diseases, with a need for a centre of gravity closer to the front line. With the frequent waves of new and emerging infections, now is as opportune time to evaluate how better to prepare for future onslaughts.

In summary, a new multidisciplinary translational research partnership emerged at KHP in response to the SARS-CoV-2 pandemic, where university and diagnostic laboratories combined with clinical academic, research and healthcare science, and service delivery teams to create a single delivery unit. It highlighted important but renewed questions about the optimal model for diagnostic innovation; laboratory configuration; and professional working linking service, research, and public health. The agenda is not new and colleagues around the world will have their own rich and recent experiences to share, no doubt with a range of views on where next? It is an open question whether our experience in South London can inform future organisational realignments, or whether it is a temporary aberration with lessons relevant only to a pandemic? For the former to be considered, we need support from the top of our structures to help capture the identity and vibrancy of these newly formed partnerships and implement positive change before things return to familiar ways.
